# Supplementary material for: Asynchronous magnetic resonance elastography: Shear wave speed reconstruction using noise correlation of incoherent waves
Source: Magn Reson Med. 2022 Oct 27;89(3):990–1001. doi: 10.1002/mrm.29502 (PMC9792433; doi:10.1002/mrm.29502)
Supplement: Supplementary file 1 — DATA S1 MATLAB code used to generate the numerical simulations of Figure 1 [file MRM-89-990-s002.zip › k-Wave/helpfiles/example_sd_focussed_detector_2D.html]

Focussed Detector In 2D Example (k-Wave)


# Focussed Detector In 2D Example

This example shows how k-Wave can be used to model the output of a focussed semicircular detector where the directionality arises from spatially averaging across the detector surface. It builds on the Homogeneous Propagation Medium and Using A Binary Sensor Mask examples.

- Open the file in the MATLAB Editor
- Run the file in MATLAB

## Contents

- Defining the sensor geometry
- Running the simulation

## Defining the sensor geometry

For large single element detectors, the directionality arises because the output is spatially averaged across the pressure field on the transducer surface. Here this effect is simulated using a focussed semicircular detector (defined using a binary sensor mask) created with `makeCircle`.

```
% define a sensor as part of a circle centred on the grid
sensor_radius = 65; % [grid points]
arc_angle = pi;     % [rad]
sensor.mask = makeCircle(Nx, Ny, Nx/2, Ny/2, sensor_radius, arc_angle);
```

## Running the simulation

To illustrate the directionality of the simulated detector, a disc shaped source (created using `makeDisc`) is first placed at the focus of the detector and then horizontally offset. A visualisation of the two simulations is given below.


The output from the simulated single element detector is then obtained by averaging the time signals recorded at each grid point within the `sensor.mask` giving a single time series output. The resulting time series for both source positions are shown below. It is clear that the detector produces a larger output for the source nearer the focus of the detector. Note, this example uses an initial value as the source of the wave, but this averaging effect will also be seen for time-varying sources.
